# Acetylated SEBS Enhanced DC Insulation Performances of Polyethylene

**DOI:** 10.3390/polym11061033

**Published:** 2019-06-11

**Authors:** Wei Dong, Xuan Wang, Zaixing Jiang, Bo Tian, Yuguang Liu, Jiaming Yang, Wei Zhou

**Affiliations:** 1 Key laboratory of Engineering Dielectrics and Its Application, Ministry of Education, College of Electrical and Electronic Engineering, Harbin University of Science and Technology, Harbin 150080, China; dongweifree@163.com (W.D.); jmyang@hrbust.edu.cn (J.Y.); 2 Institute of Radiation Medicine, China Academy of Medical Science & Peking Union Medical college, Tianjin 300192, China; zhouwei5605@163.com; 3 Department of Polymer Science and Technology, School of Chemical Engineering and Technology, Harbin Institute of Technology, Harbin 150001, China; 4 Technical Physics Institute of Heilongjiang Academy of Sciences, Harbin 150086, China; tianbo_0627@163.com (B.T.); ygliu_63@163.com (Y.L.)

**Keywords:** polyethylene, voltage stabilizer, insulation material, DC breakdown strength, space charge

## Abstract

Acetophenone can significantly improve the dielectric properties of polyethylene (PE) insulation materials. However, it easily migrates from the PE due to its poor compatibility with the material, which limits its application. In this paper, the functional units of acetophenone were modified in polystyrene-b-poly(ethylene-*co*-butylene)-b-polystyrene (SEBS) by an acetylation reaction, and SEBS was used as the carrier to inhibit the migration of acetophenone. The number of functional units in the acetylated SEBS (Ac-SEBS) was measured by ^1^H NMR and the effect of the acetylation degree of SEBS on its compatibility with PE was studied. Meanwhile, the effects of Ac-SEBS on PE’s direct current (DC) breakdown strength and space charge accumulation characteristics were investigated. It is demonstrated that Ac-SEBS can significantly improve the field strength of the DC breakdown and inhibit the accumulation of space charge in the PE matrix. This work provides a new approach for the application of aromatic compounds as voltage stabilizers in DC insulation cable materials.

## 1. Introduction

Compared to alternating current (AC) transmission, direct current (DC) transmission has several drawbacks, such as the need for additional conversion equipment and maintenance difficulties. However, DC transmission has the advantages of large capacity, good stability and low power consumption, and is suitable for long-distance high-power transmission [1,2,3,4]. Polyethylene (PE) insulation materials have been widely used in high-voltage DC cables due to their excellent electrical properties and good processing properties [5,6]. With the continuous increase of electricity consumption in cities and the widespread use of urban underground cables, how to further improve the transmission capacity and service life of DC cables has become a topic of great interest in the research field of cables. The performance of insulating materials has always been an important factor restricting the development of DC cables. Increasing the breakdown strength of insulating materials and suppressing the accumulation of space charge are the key issues to resolve in order to increase the transmission capacity and service life of cables [7,8,9,10].

Voltage stabilizers and inorganic nano-fillers have been widely reported to improve the breakdown strength and suppression of space charge accumulation of insulation materials [11,12,13,14,15,16,17]. Although nanoparticles are easy to agglomerate in polymers, agglomeration in polymer nanocomposites can be prevented by controlling the interface chemistry [18,19,20,21,22]. Acetophenone (AP) and its derivatives have high electron affinity, allowing them to capture high-energy electrons and weaken the electron energy, thus inhibiting the destruction of the PE molecular chain by high-energy electrons. As voltage stabilizers composited with PE, they can substantially improve the breakdown strength and suppress the space charge of PE [23,24]. However, the small, polar organic molecules have poor compatibility with the non-polar PE matrix and, consequently, easily migrate, which affects the long-term stability of the materials [25,26]. Jarvid and Englund et al. [27,28,29,30] reported that the compatibility of PE could be enhanced by introducing alkyl side chains into the voltage stabilizer molecules, which further increased the stability of the composite. Nevertheless, this method still cannot fundamentally inhibit the migration of the voltage stabilizer. Hui Zhang et al. [31,32] found by theoretical calculations that voltage stabilizers could be grafted onto the PE molecular chains during the cross-linking process of PE, which inhibited the migration of the voltage stabilizer to some extent and were able to improve the dielectric properties of PE. In our previous work, we developed a graftable acetophenone voltage stabilizer [33]. Studies have shown that grafting can significantly inhibit the migration of a voltage stabilizer, but the grafting efficiency of small-molecule voltage stabilizers onto PE is problematic, as some voltage stabilizers remain in the form of small molecules in the PE matrix and adversely affect the long-term service life of PE insulation materials.

Polystyrene-b-poly(ethylene-*co*-butylene)-b-polystyrene (SEBS) exhibits good compatibility with PE and has been studied as a dispersant and toughening agent for the modification of polyolefin insulation materials [34,35,36,37]. Ma et al. [38] studied the electrical, mechanical, and growth behavior of a water tree in SEBS/crosslinked polyethylene (XLPE) composites. The results show that the addition of SEBS can significantly improve the inhibition effect and mechanical properties of an XLPE water tree, but the breakdown strength is decreased by some degree. If SEBS is combined with a voltage stabilizer, not only can the small-molecule voltage stabilizers be inhibited from migrating in the PE matrix, but the voids generated in the PE insulating material due to volatilization during processing and crosslinking of the small-molecule voltage stabilizers can also be avoided. In this study, acetophenone structural units were produced in SEBS by the acetylation reaction, and PE was modified by acetylated SEBS (Ac-SEBS), The acetophenone functional unit has the effect of voltage stabilization, which improves the DC breakdown strength of the composites. As a polar group, the acetophenone functional unit can introduce deep traps in the composites and inhibit the accumulation of space charge in the composites.

## 2. Materials and Methods

### 2.1. Materials

Low density polyethylene (LDPE) was purchased from China petrochemical group co., Ltd. (Beijing, China). Analytical grade of Dichloromethane, methanol, and heptane were purchased from Tianjin fuyu fine chemical co., Ltd. (Tianjing, China). Analytical grade of Acetyl chloride and anhydrous aluminum chloride (AlCl_3_) were purchased from Aladdin industrial corporation. (Shanghai, China).

### 2.2. Preparation of Ac-SEBS

**Scheme 1:**
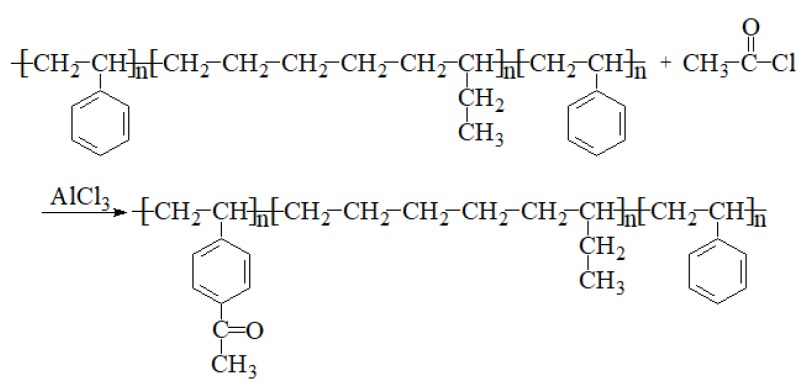
Synthesis of Ac-SEBS.

Ac-SEBS was prepared as illustrated in Scheme 1. A quantity of 15 g SEBS (0.043 mL styrene repeating unit) was added to a 250 mL round-bottom flask, then 200 mL dichloromethane was added, and the mixture was stirred at room temperature until the SEBS had been completely dissolved. Then, a certain amount of acetyl chloride solution (0.1, 0.4, 0.6, 1.0 and 2.0 mL) was added and stirred evenly; the degree of acetylation of SEBS was controlled by regulating the addition quantity of acetyl chloride. After that, 1.4 g of anhydrous AlCl_3_ (0.01 mol) powder was added and stirred at room temperature for 4 h. The reaction solution was slowly poured into the methanol solution under stirring. Next, the formed white flocculent precipitates were filtered and dried at 60 °C in a vacuum oven until their weight did not change. The dried products were again dissolved in dichloromethane, precipitated in methanol solution, filtered, and dried; this was repeated three times to obtain the Ac-SEBS for subsequent experiments.

### 2.3. Preparation of Ac-SEBS/PE

Ac-SEBS and PE were proportionally added to the torque rheometer (Harbin, China) and melt blended at a speed of 60 rpm at 150 °C for 15 min to obtain the Ac-SEBS/PE composite. Samples of different thicknesses according to the test requirements were prepared using a flat vulcanizer (Qingdao, China) at a temperature of 120 °C and a pressure of 15 MPa.

### 2.4. Fourier Transform Infrared Spectroscopy (FTIR)

SEBS and Ac-SEBS were pressed into thin slices by a flat vulcanizer. ATR-FTIR spectroscopy of SEBS and Ac-SEBS were performed using a Nicolet iS5 spectrometer (Madison, USA) and spectra from 400 to 4000 cm^−1^ were recorded. The resolution was 4 cm^−1^ with a total of 32 scans.

### 2.5. Nuclear Magnetic Resonance Hydrogen (^1^H NMR) spectroscopy

The ^1^H NMR spectra were determined using a Bruker AVANCE III (Karlsruhe, Germany) at 400 MHz (^1^H) using CDCl_3_ as a solvent.

### 2.6. Scanning Electron Microscopy (SEM)

The morphology of the Ac-SEBS/PE mixtures was observed at a low acceleration voltage of 5 kV using the Hitachi SU8020 (Tokyo, Japan) cold field emission high-resolution SEM. The sample was fractured in liquid nitrogen, and the Ac-SEBS phase was dissolved in *n*-heptane at room temperature. After washing and drying, the sample was attached to the sample stage, and after the surface underwent gold spraying treatment, the morphology of the sample was observed.

### 2.7. Differential Scanning Calorimetry (DSC)

The thermal properties of the samples were measured using a Mettler Toledo DSC822e (Greifensee, Switzerland). Each sample was first heated from 50 to 150 °C at 10 °C/min and held for 5 min to erase the thermal history, then cooled to 50 °C and reheated to 150 °C. The crystallization curve was recording during the cooling process. The melting curve was recording during the second heating cycle. The measurements were carried out under a nitrogen atmosphere. The degree of crystallinity of samples were calculated by Equation (1) [39]:

(1) $$\mathrm{Xc}=\frac{\Delta H_{m}}{\Delta H_{m,PE}^{\infty }}\times \frac{1}{\varphi \left(PE\right)}\times 100\;$$

where $\Delta H_{m}$ is the observed melting enthalpy of the PE phase in the sample obtained from DSC, $\Delta H_{m,PE}^{\infty }$ is 293 J/g for 100% crystalline PE, and φ(*PE*) is the PE weight fraction in the sample.

### 2.8. Samples Extraction

The samples were placed in copper mesh bags and then placed in the Soxhlet extractor (Taizhou, Jiangsu, China). Distilled water was added into a flat-bottomed flask, which was heated to boiling through a heating jacket. The samples were extracted for 24 h. After extraction, the samples were dried at 60 °C in a vacuum oven for 4 h.

### 2.9. DC Breakdown Strength

A cylindrical electrode was used for the breakdown test. The diameters of the high-voltage electrode and the ground electrode were 25 and 50 mm, and the edges of both electrodes had 5 mm rounded corners. A circular sample with a thickness of 100 μm and a diameter of 80 mm was used. During the experiment, the sample and the electrode were immersed in the silicone oil. The DC breakdown test adopted a linear boosting method by applying a DC voltage to the sample at a boost rate of 0.5 kV/s until the sample break down, reading the voltage value when it break down, and calculating the DC breakdown strength according to the formula *E* = *U*/*d*, where *U* is the voltage at which the sample broke down, *d* is the thickness of the sample, and *E* is the breakdown strength of the sample. The breakdown strength was calculated using the Weibull distribution function, and the breakdown strength corresponding to a cumulative failure probability of 63.2% was taken as the characteristic breakdown strength of the material.

### 2.10. Thermal Stimulated Current (TSC)

The thermal stimulated current method was used to test the charge trap characteristics. Aluminum electrodes were plated on the surface of the samples; the diameter of the aluminum electrodes was 25 mm and the samples were 0.1 mm in thickness. The samples were heated to 23 °C in a vacuum environment and then were electrically polarized by applying 30 kV/mm DC high voltage for 30 min, and the samples were then rapidly cooled to below −50 °C so that all kinds of charge carriers were “frozen”. The samples were heated linearly under a constant heating rate of 3 °C/min, and the short-circuit current was measured using a 6517b electrometer (Keithley Instrument Inc., Cleveland, OH, USA).

### 2.11. Space Charge

The space charge characteristics of PE, SEBS/PE, and Ac-SEBS/PE were tested using a Pulsed Electro-Acoustic (PEA) technique (Shanghai, China). The samples were 50 mm in length and 0.25 mm in thickness with aluminum electrodes of 25 mm in diameter vacuum-deposited on both sides. The test conditions were as follows: polarization temperature was 25 °C, polarization electric field was 30 kV/mm, pulse voltage was 400 V, pulse width was 8 ns, and input impedance was 1 MΩ. After the samples were polarized under pressure for 30 min at an electric field strength of 30 kV/mm, their ends were short-circuited for 30 min, and changes in the space charge in the sample under pressure and short-circuit conditions measured.

## 3. Results

### 3.1. FTIR Spectroscopy

**Figure 1:**
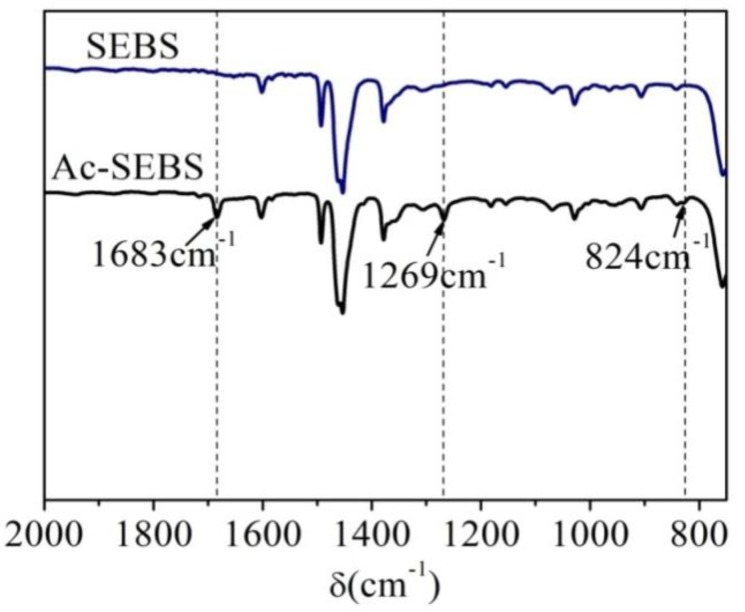
Fourier Transform Infrared Spectroscopy (FTIR) spectra of polystyrene-b-poly(ethylene-*co*-butylene)-b-polystyrene (SEBS) and Ac-SEBS.

Figure 1 shows the infrared spectra of SEBS and Ac-SEBS. The absorption peaks of SEBS and Ac-SEBS at 1601, 1491, and 1452 cm^−1^ are the characteristic absorption peaks of skeleton deformation vibration of the benzene ring, while the absorption peak at 1378 cm^−1^ corresponds to the vibration of the C–H on the methyl group. Ac-SEBS has three additional absorption peaks at 1683, 1269, and 824 cm^−1^, where the absorption peak at 1683 cm^−1^ is the C=O stretching vibration peak; the peak at 1269 cm^−1^ stems from the vibration of the aromatic ketone skeleton; and the weak absorption peak at 824 cm^−1^ is the characteristic absorption peak of out-of-plane bending vibration of C–H in the benzene ring with disubstituted. Infrared spectrum analysis shows that the acetylation reaction produced acetophenone functional units in the styrene block of SEBS.

### 3.2. Determination of the Acetylation Degree of SEBS

**Figure 2:**
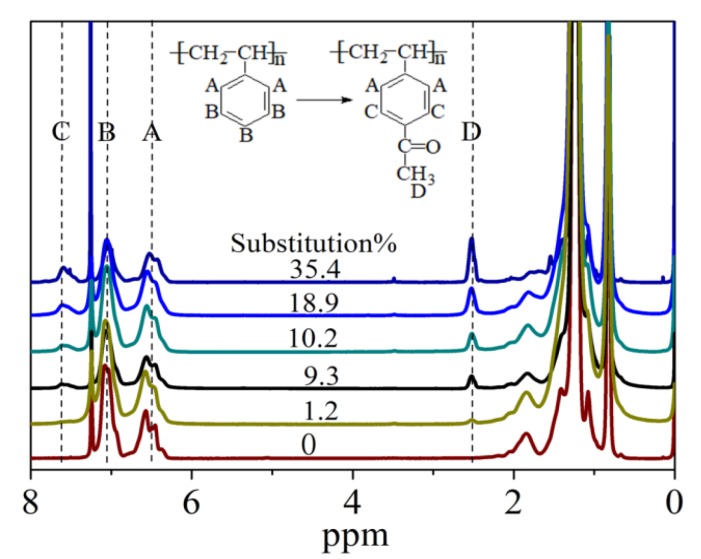
Nuclear Magnetic Resonance Hydrogen (NMR) hydrogen spectra of SEBS with different Acetylation Degrees.

Figure 2 shows the ^1^H NMR spectra of SEBS and Ac-SEBS with different degrees of acetylation. The proton resonance of the benzene rings in SEBS and Ac-SEBS generally occurs in three regions (A, 6.3–6.8 ppm; B, 6.8–7.2 ppm C, 7.35–7.8 ppm). Region A corresponds to the absorption peak of the neighboring proton of the benzene ring in SEBS and Ac-SEBS; Region B corresponds to the absorption peak of the meta and para protons of the benzene ring in SEBS; and Region C corresponds to the peak formed by the migration of the absorption peaks of two protons adjacent to the carbonyl group towards the high field following the π–π conjugate between the acetylated benzene ring and carbonyl. The new peak appearing at 2.4–2.6 ppm is the proton peak of CH_3_ of the acetyl group. Since the area of the resonance peak in the ^1^H-NMR spectrum is proportional to the number of protons that produce the peak, we can calculate the degree of acetylation of SEBS from the ratio of the proton peak areas. The number of protons represented by A does not change with the acetylation reaction of SEBS, whereas the number of protons represented by D increases with the degree of acetylation. The ratio of the area of peak A to the area of peak D can be used to calculate the degree of acetylation of SEBS. The degree of acetylation of the sample was therefore calculated using Equation (2):

(2) $$sub\%=\frac{2\times A_{D}}{3\times A_{A}}\times 100\%$$

where *A_A_* and *A_D_* are the areas of the proton peaks A and D, respectively. The respective degree of acetylation of the samples were calculated to be 35.4%, 18.9%, 10.2%, 9.3%, and 1.2%.

### 3.3. Dispersion and Morphology

**Figure 3:**
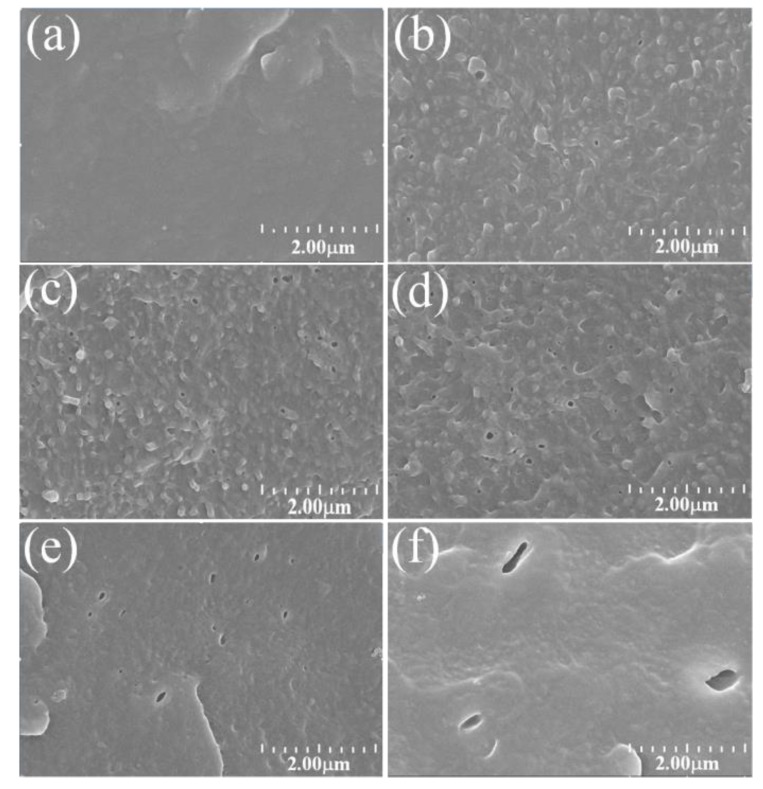
SEM images of Ac-SEBS/PE composites with different acetylation degrees of SEBS: (**a**): polyethylene (PE), (**b**): 1.2%; (**c**): 9.3%; (**d**): 10.2%; (**e**): 18.9%; (**f**): 35.4%.

The dispersibility and particle size of Ac-SEBS in the PE matrix are the key factors controlling the dielectric properties of Ac-SEBS/PE composites. Figure 3 shows SEM images of PE and 2% Ac-SEBS/PE composites with SEBS acetylated by 1.2%, 9.3%, 10.2%, 18.9%, and 35.4%. The holes in Figure 3 are a result of etching the Ac-SEBS. As can be seen from the Figure 3, Ac-SEBS/PE has the typical “sea–island” structure, where the Ac-SEBS phase is the island and the PE phase is the sea. When the degree of acetylation is 1.2%, 9.3%, or 10.2%, Ac-SEBS is uniformly distributed in PE with a particle size of 100–200 nm. As the degree of acetylation increases, the pores in the composite material change from round to strip shaped. Additionally, the particle size increases due to poor dispersion in PE because of the interaction of polar groups in Ac-SEBS. This result indicates that the distribution and the particle size of the Ac-SEBS phase in the PE phase can be controlled by the degree of acetylation of SEBS.

### 3.4. Crystallization Characteristics

**Figure 4:**
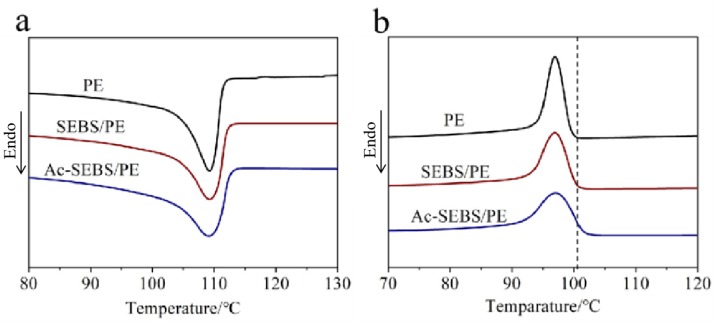
(**a**) Melting curves and (**b**) crystallization curves of PE, SEBS/PE, and Ac-SEBS/PE.

Figure 4 demonstrates the melting and crystallization curves of PE, 2% SEBS/PE, and 2% Ac-SEBS/PE, degree of acetylation of Ac-SEBS is 9.3%. Table 1 summarizes the initial crystallization temperature, melting enthalpy, and crystallinity of the samples. It can be seen from Table 1 and Figure 4b that the introduction of SEBS and Ac-SEBS results in a decrease in crystallinity and a broadening of the crystallization peak as a result of SEBS and Ac-SEBS inhibiting the movement of the PE molecular segments in the material. Consequently, the PE becomes difficult to crystallize, the molecular chain is insufficiently arranged in the crystallization, the grain size distribution is broadened, and, finally, the crystallinity is reduced. It can be seen from Table 1 that the initial crystallization temperatures of SEBS/PE and Ac-SEBS/PE increase, indicating that SEBS and Ac-SEBS act as nucleating agents in the crystallization process. Since Ac-SEBS contains polar groups which have a more obvious effect as the nucleating agent, its crystallinity is significantly higher than that of SEBS/PE.

### 3.5. Stability of Ac-SEBS/PE

**Figure 5:**
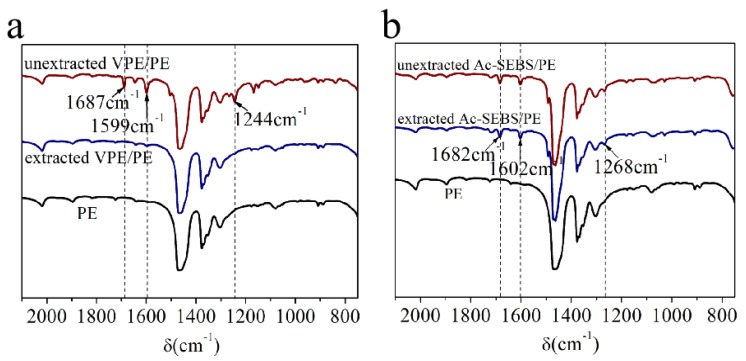
Infrared spectrum pre-and post-extraction of 1-(4-vinyloxy)phenylethenone (VPE/PE) and Ac-SEBS/PE. (**a**) VPE/PE, (**b**) Ac-SEBS/PE.

The stability of the samples was analyzed by comparing the infrared spectra of the samples before and after extraction. Figure 5a is infrared spectrum pre-and post-extraction of 1-(4-vinyloxy)phenylethenone (VPE)/PE blends. It can be seen from Figure 5a that the characteristic absorption peaks of VPE at 1687 cm^−1^, 1599 cm^−1^ and 1244 cm^−1^ can be seen in the infrared spectra of VPE/PE sample before extraction. The characteristic absorption peaks of VPE disappeared in the infrared spectra of VPE/PE sample after extraction. This indicates that the free VPE was removed from PE after extraction. As shown in Figure 5b, the characteristic absorption peaks of AP in Ac-SEBS/PE at 1682 cm^−1^, 1602 cm^−1^ and 1268 cm^−1^ hardly changed pre-and post-extraction. This proves that AP is stable in Ac-SEBS/PE.

### 3.6. DC Breakdown Strength of Ac-SEBS/PE

**Figure 6:**
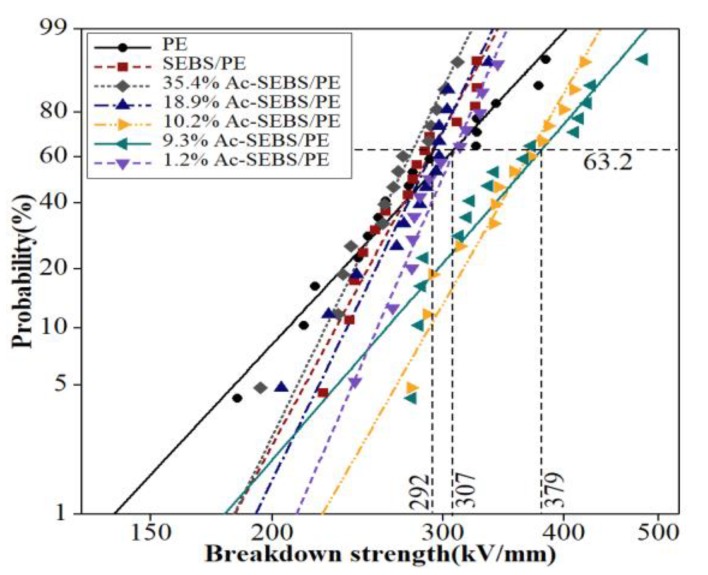
Weibull plots of the DC breakdown strength of PE, SEBS/PE, and Ac-SEBS/PE.

The DC breakdown strength values of PE, SEBS/PE, and Ac-SEBS/PE with different degrees of acetylation are shown in Figure 6, and the shapes and DC breakdown strengths are summarized in Table 2. The addition of SEBS and Ac-SEBS in samples is 2 wt %. From Figure 6, we can see that the breakdown strength of PE is 307 kV/mm and the breakdown strength of SEBS/PE is 292 kV/mm; this shows that the introduction of SEBS reduces the breakdown strength of PE by 5%. The effect of Ac-SEBS on the breakdown strength of PE first increases and then decreases with increasing acetylation degree. The reason for this is that while the degree of acetylation of SEBS is low, the Ac-SEBS phase can still disperse uniformly in the PE phase with smaller particle size. At the same time, acetylation reactions introduce acetophenone functional units into SEBS, which can absorb high-energy electrons and avoid the breakdown of PE molecular chains under high-energy electron bombardment, thereby improving the breakdown strength of PE. When the degrees of acetylation are 9.3% and 10.2%, the breakdown strengths of Ac-SEBS/PE are 379 kV/mm and 370 kV/mm, respectively, which are obviously higher than that of PE. An acetylation degree of 9.3% has the best effect, the breakdown strength is increased by 23.2%. With increasing acetylation degree, the compatibility of Ac-SEBS with PE becomes poorer, which degrades the breakdown strength of PE. When the acetylation degree reaches 18.9% or 35.4%, the breakdown strength of Ac-SEBS/PE decreases to even lower than that of PE.

**Figure 7:**
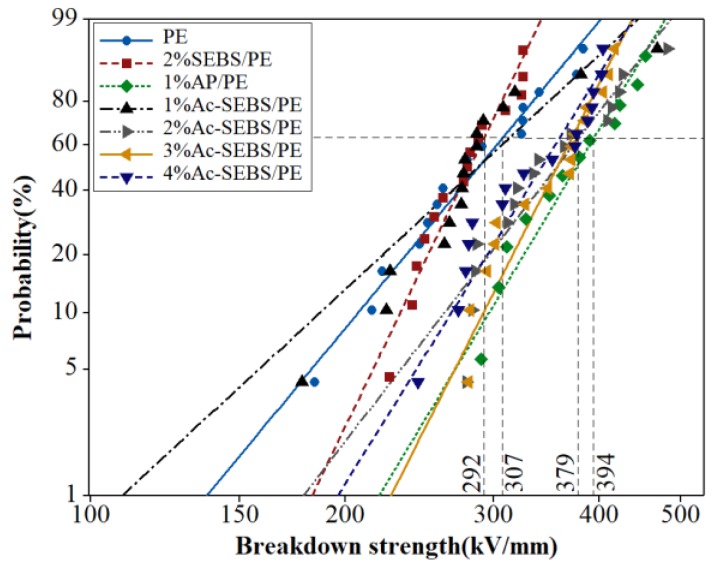
Weibull plots of the DC breakdown strength of Ac-SEBS/PE with different contents of Ac-SEBS.

As acetylation degree of 9.3% has the best effect, we further investigate the effect of Ac-SEBS content on DC breakdown strength of PE under acetylation degree of 9.3%. The DC breakdown strengths of PE, AP/PE, SEBS/PE and Ac-SEBS/PE with different proportions are shown in Figure 7, and the shapes and DC breakdown strengths are summarized in Table 3. The different proportions of Ac-SEBS can improve the breakdown strength of PE; the result shows that the addition of 2% Ac-SEBS has the best effect, and the breakdown strength can be increased by 23.2%. As the amount of Ac-SEBS increases, the breakdown strength shows a downward trend; this is because with increasing Ac-SEBS, the particle size of the Ac-SEBS phase in the PE phase will gradually increase, so the DC breakdown strength of Ac-SEBS/PE decreases. By comparing the DC breakdown strength of PE, 1% AP/PE and 2% Ac-SEBS/PE, Ac-SEBS improves DC breakdown strength of PE slightly less than AP, which is ascribed to the low efficiency of introducing AP into PE by Ac-SEBS.

### 3.7. Trap Level Distribution

**Figure 8:**
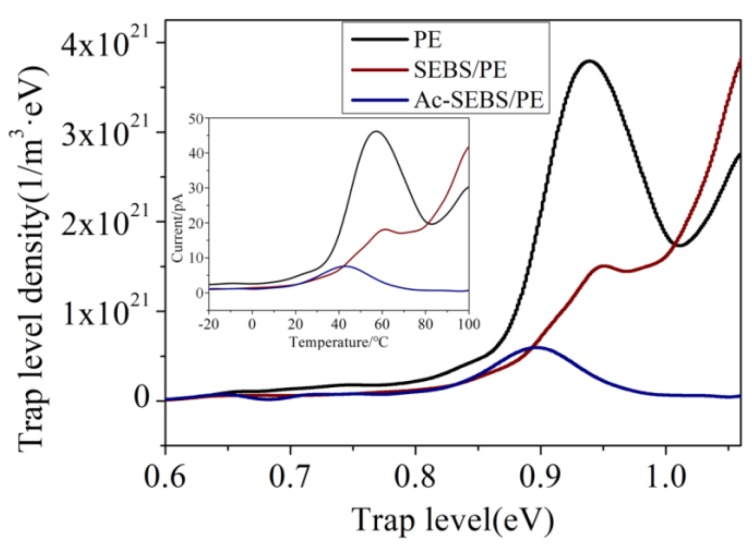
Thermal Stimulated Current (TSC) spectra of PE, SEBS/PE and Ac-SEBS/PE.

The trap level distributions of PE, 2% SEBS/PE and 2% Ac-SEBS/PE are shown in Figure 8. The acetylation degree of Ac-SEBS is 9.3%. It can be seen from Figure 8 that the peak thermal stimulation current of PE appears near 60 and 100 °C. These trapped charges mainly come from the structural defects of PE. The TSC current at 60 °C is derived from the release process of the charge that is trapped by the interface between crystalline and amorphous regions and the structural defects between lamellae. This trap depth is relatively shallow. The TSC current at 100 °C comes from the release process of the charge that is trapped by the structural defects in the crystal region; this trap depth is deeper. Comparing the TSC current results of PE and SEBS/PE, the TSC current value of SEBS/PE near 60 °C is significantly lower than that of PE, while the TSC current value near 100 °C is significantly increased, indicating that the deep trap density in SEBS/PE is increased. It can be seen from the DSC test results that the introduction of SEBS reduces the crystal regularity of PE, thus introducing more structural defects in the crystal region, that is, increasing the deep trap density. The presence of deep traps can significantly inhibit the migration of injected charges into the interior of PE. From the test results, it can be seen that the space charge in SEBS/PE is more concentrated near the electrode, while the PEA test results of PE show that the space charge is injected into the inside of the sample. This is because the density of deep traps in PE is small, and the space charge is trapped in a relatively shallow trap; thus, it continuously migrates into the interior of the PE, forming a large amount of space charge. We can see that increasing the deep trap density is helpful in inhibiting the space charge. In addition, SEBS contains weakly polar polystyrene blocks, and it is also possible to produce uniformly distributed traps in PE, which have a certain space charge suppression effect. The TSC curve of Ac-SEBS/PE was different from those of PE and SEBS/PE; it showed only a small current release peak at 40 °C, and no significant TSC current peak appeared at other temperatures. From the results of PEA and DSC tests, it can be seen that Ac-SEBS/PE has stronger space charge suppression ability, and the damage by Ac-SEBS to the crystallization regularity of PE is more obvious than SEBS, so the current release peak should be more obvious near 100 °C. In recent years, many studies have reported that polar groups can introduce deep traps into polyolefin materials to form very strong space charge inhibition ability. Ac-SEBS’s modified acetophenone unit carries strong polar groups and has the possibility of forming deep traps. It can also be inferred from the TSC test results that the release temperature of charge captured by deep traps in Ac-SEBS/PE is probably higher than the melting point of PE, so it was not measured in the TSC test.

### 3.8. Space Charge Characteristics

**Figure 9:**
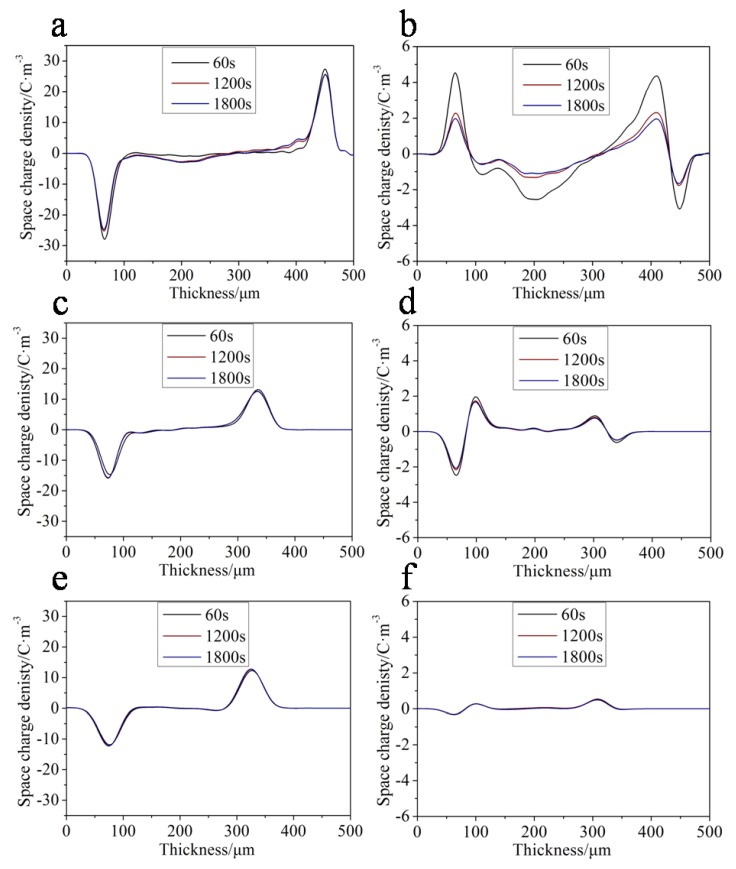
Space charge distributions (**a**) PE, (**c**) SEBS/PE, (**e**) Ac-SEBS/PE) and short circuit conditions (**b**) PE, (**d**) SEBS/PE, (**f**) Ac-SEBS/PE.

Space charge accumulation is an important factor causing insulation failure in DC cables; therefore, the space charge accumulation in insulation should be as low as possible. Figure 9 shows the space charge distribution and short circuit conditions of PE, 2% SEBS/PE, and 2% Ac-SEBS/PE. It is noted from Figure 9a that a large number of charges are injected near both the cathode and anode in PE. In comparison, Figure 9c,e show that the numbers of injected charges in SEBS/PE and Ac-SEBS/PE are significantly lower than that in PE, especially in Ac-SEBS/PE, where almost no charges exist near the cathode and anode.

The space charge distribution under short circuit of PE, SEBS/PE, and Ac-SEBS/PE are shown in Figure 9b,d,f. We can see from Figure 9b that the peak densities of the positive and negative space charges of PE are and 3.04 C/m^3^; after 30 min, they are reduced to 1.97 and 1.66 C/m^3^. It can be seen from Figure 9d that the amount of charge in the SEBS/PE near the cathode and anode is significantly reduced: the peak densities of space charge are 1.94 and 0.62 C/m^3^, but after 30 min, they are reduced to 1.66 and 0.47 C/m^3^. The decrease in the charge density is less than that of PE, which indicates that the trap depth in SEBS/PE is deeper than that in PE. Figure 9f shows that Ac-SEBS/PE only has a small amount of space charge near the cathode: the peak densities of space charge are 0.27 C/m^3^, and after 30 min, the densities of space charge hardly decrease. This indicates that Ac-SEBS/PE has the deepest trap depth and the best space charge suppression effect. This is because Ac-SEBS contains carbonyl polar groups, and more deep traps are introduced into the material. Thus, the charge injected by the electrode is locked near the electrode to form an independent electric field, which reduces the effective electric field near the electrode and increases the barrier of charge injection, so the space charge in the material is significantly suppressed.

## 4. Conclusions

In this study, Ac-SEBS was used to improve the DC breakdown strength and the space charge suppression ability of PE. It was found that Ac-SEBS can be uniformly distributed in the PE matrix at 100–200 nm scale by adjusting the degree of acetylation of Ac-SEBS. Due to Ac-SEBS containing acetophenone functional units, it can inhibit the destruction of the PE molecular chain by high-energy electrons and significantly improve the DC breakdown strength of PE, DC breakdown strength of PE increases by 23.2%. Ac-SEBS in PE plays the role of a nucleating agent that makes PE spherulites smaller and irregular, which increases the boundary of spherulites and introduces more traps into the PE matrix; meanwhile, the polar groups carried by Ac-SEBS form deep traps with a uniform distribution in the PE matrix, and space charge accumulation is inhibited.

## Figures and Tables

**Table 1:** Summary of Differential Scanning Calorimetry (DSC) data of PE, SEBS/PE, and Ac-SEBS/PE.

Sample	Initial Crystallization Temperature/°C	Melting Enthalpy/J·g^−1^	Crystallinity/%
PE	100.5	102.9	35.1
SEBS/PE	101.8	91.8	31.3
Ac-SEBS/PE	103.3	100.3	34.2

**Table 2:** DC characteristic breakdown strengths and shape parameters of different acetylation degrees.

Sample	Acetylation Degree of Ac-SEBS	Shape	DC Breakdown Strength/kV mm^−1^
PE	—	5.70	307
2% SEBS/PE	—	9.80	292
2% Ac-SEBS/PE	1.2%	12.26	308
2% Ac-SEBS/PE	9.3%	6.12	379
2% Ac-SEBS/PE	10.2%	9.28	370
2% Ac-SEBS/PE	18.9%	10.87	293
2% Ac-SEBS/PE	35.4%	10.85	279

**Table 3:** DC characteristic breakdown strength and shape parameter of different Ac-SEBS contents.

Sample	Shape	DC Breakdown Strength/kV mm^−1^
PE	5.70	307
1% AP/PE	7.85	394
2% SEBS/PE	9.80	292
1% Ac-SEBS/PE	4.35	313
2% Ac-SEBS/PE	6.12	379
3% Ac-SEBS/PE	9.31	371
4% Ac-SEBS/PE	7.61	359
